# A pattern learning-based method for temporal expression extraction and normalization from multi-lingual heterogeneous clinical texts

**DOI:** 10.1186/s12911-018-0595-9

**Published:** 2018-03-22

**Authors:** Tianyong Hao, Xiaoyi Pan, Zhiying Gu, Yingying Qu, Heng Weng

**Affiliations:** 1 School of Information Science and Technology, Guangdong University of Foreign Studies, Guangzhou, China; 2 School of Computer, South China Normal University, Guangzhou, China; 3 School of Business, Guangdong University of Foreign Studies, Guangzhou, China; 4 The Second Affiliated Hospital, Guangzhou University of Chinese Medicine, Guangzhou, China

**Keywords:** Temporal expression identification, Heuristic rule, Pattern generation, Clinical texts, Heterogeneous

## Abstract

**Background:**

Temporal expression extraction and normalization is a fundamental and essential step in clinical text processing and analyzing. Though a variety of commonly used NLP tools are available for medical temporal information extraction, few work is satisfactory for multi-lingual heterogeneous clinical texts.

**Methods:**

A novel method called TEER is proposed for both multi-lingual temporal expression extraction and normalization from various types of narrative clinical texts including clinical data requests, clinical notes, and clinical trial summaries. TEER is characterized as temporal feature summarization, heuristic rule generation, and automatic pattern learning. By representing a temporal expression as a triple <*M, A*, *N*>, TEER identifies temporal mentions *M*, assigns type attributes *A* to *M*, and normalizes the values of *M* into formal representations *N*.

**Results:**

Based on two heterogeneous clinical text datasets: 400 actual clinical requests in English and 1459 clinical discharge summaries in Chinese. TEER was compared with six state-of-the-art baselines. The results showed that TEER achieved a precision of 0.948 and a recall of 0.877 on the English clinical requests, while a precision of 0.941 and a recall of 0.932 on the Chinese discharge summaries.

**Conclusions:**

An automated method TEER for multi-lingual temporal expression extraction was presented. Based on the two datasets containing heterogeneous clinical texts, the comparison results demonstrated the effectiveness of the TEER method in multi-lingual temporal expression extraction from heterogeneous narrative clinical texts.

## Background

The popularity of Electronic Medical Record (EMR) provides opportunities and challenges to accelerate clinical science using large scale of practical clinical data in less expense [1]. As a result, a large amount of unstructured clinical texts is generated such as clinical discharge summaries and progress notes, which contain a variety of temporal expressions and medical concepts [2]. Taking a clinical discharge summary as an example, it is a type of clinical reports by physicians or other medical professionals at the end of a hospital stay or series of treatments. A summary text outlines a patient’s admitting diagnosis, diagnostic findings, diagnostic procedures performed, therapy received while hospitalized, and clinical course during hospitalization, prognosis, and plan of action upon discharge with stated time to follow up [3]. Consequently, temporal expressions usually are involved in sequential events and patients’ phenotype value variations with time. Therefore, it is highly meaningful to extract temporal expressions and their associated diagnosis procedure actions as well as patient phenotypes for deep analysis [4]. Similarly, automatic processing and formulation of clinical data requests is desired to improve the labor-intensive, time-consuming process of data requests translation into executable database requests [5, 6]. However, the identification of temporal expressions is one of major bottlenecks to the automatic data request formulation. Our previous analysis on 400 actual data requests submitted to the Columbia University Medical Center (CUMC)‘s clinical data warehouse showed that about 64% of the data requests contain temporal expressions [1].

Temporal expression extraction is also essential to an extensive scope of Natural Language Processing (NLP) applications, e.g., question understanding and text comprehension, since the extraction plays an unique role in the analysis of chronological events [7, 8]. Narrative clinical texts are different from other types of unstructured texts. They commonly contain a large number of International Classification of Diseases (ICD), Admit Discharge Transfers (ADT), Current Procedural Terminology (CPT) codes, concept abbreviations, etc., increasing the complexity of texts dramatically [1]. For example, the ICD code “*002.2*” may cause a confusion with a temporal expression “*2002.2*”. A large amount of quantitative expressions exist and may also incur wrong identification [9, 10]. Moreover, a wide range of representations in particularly special temporal expression formats, e.g., “*May 30 = 04*” and “*2 + 月*”, causing the texts even more difficult to process. In addition, the texts frequently contain noise data, e.g., incorrect spelling “*2 moths*”. As a result, a number of commonly used NLP tools have been applied for medical temporal expression extraction, but few work works well for multi-lingual heterogeneous narrative clinical texts.

To that end, we develop a new method – Temporal Expression ExtractoR (TEER). TEER integrates heuristic rule generation and automatic pattern learning to extract temporal expressions from various types of narrative clinical texts, e.g., clinical data requests, discharge summary texts, etc. In TEER, a list of structural patterns are automatically learned from a part of annotated texts. After that, the patterns are validated by calculating their confidence values through matching with more annotated texts. Eventually, TEER utilizes achieved patterns with summarized heuristic rules to extract candidate temporal expressions and filter out irrelevant ones.

Our experiments used two datasets: 1) 400 actual clinical data requests from CUMC’s data warehouse. The requests were labelled by three human annotators. 100 request texts were used for training and the other 300 were used for testing. 2) The unstructured discharge summaries from the electronic medical records (EMR) of 1459 actual patients with breast cancer disease, who received medical treatment (operation) from a 3A hospital in mainland China. These discharge summaries were also manually labelled by three human annotators. 276 patient records were randomly selected and used for training and 134 records were used for testing. We used 6 baseline methods including CMedTEX, GUTime, and HeidelTime for performance comparison. The results presented that our method TEER outperformed all the baselines and demonstrated that the TEER method was effective in extracting temporal expressions from multi-lingual heterogeneous clinical texts.

### Related work

In response to the need of temporal expression extraction, an open evaluation challenge-TempEval for temporal expression identification, was held in 2007 [8], 2010 [11], 2013 [12], 2015 [13], 2017 [14], resulting in the wide adoption of a number of systems. HeidelTime [7], an instance of the systems, outperformed the other systems the English temporal expression identification and normalization task of the TempEval 2 challenge. TempEval challenge released official guidelines for annotating temporal expressions in the challenges. For example, the guideline for English text annotation in TempEval 2010 consisted of nouns, proper nouns, noun phrases, adjectives, adjective phrases, adverbs, and adverb phrases.

Targeting at temporal expression extraction, TempEx recognized temporal expressions and normalized them using the TIMEX2 standard. Both absolute time (e.g., *May 7, 2017*) and relative time (e.g., *last weekend*) could be identified by TempEx through the way of local context. GUTime further enhanced the capabilities. Based on the idea of utilizing a reference time, the method identified and annotated lexical triggers such as *yesterday* and phrase triggers such as *last year*. Temporal extraction gained more attention since 2010. As the result, more progressive methods about temporal expression extraction were developed, e.g., HeidelTime. Nevertheless, all the methods focused on newspaper and narrative texts primarily, without testing on medical texts [1].

For clinical temporal expression extraction, Informatics for Integrating Biology & the Bedside (i2b2) NLP Challenge devoted on temporal relation identification in medical narratives for EMR data records. The challenge also offered a corpus containing clinical discharge summaries with human annotations of events and temporal expressions for research communities. The corpus was widely applied to the development and evaluation of temporal expression and event identification methods [15]. The challenge tried to evaluate different submitted methods on: 1) temporal expressions including date, time, duration, or frequency types, 2) clinical events containing medical concepts such as treatments, and events related to the clinical timeline of patients, e.g., admissions, transfers among departments, and 3) temporal relations between temporal expressions and clinical events.

Clinical TempEval 2015 concentrated on the method competition for timeline extraction and annotation for the medical domain. The challenge included six different tasks. The Task 12 (clinical TempEval) of SemEval-2017 succeeded Clinical TempEval [16] and the past i2b2 temporal challenge [17] directly. The Clinical TempEval focused on clinical timeline extraction and understanding for clinical narratives, basing on the THYME corpus with temporal annotations [18]. 16 teams participated in TempEval 2017 [19].

There are a number of research and systems for English temporal expression extraction from clinical texts. Sohn et al. reported a hybrid method to detect temporal information using regular expression matching and matching learning [15]. A comprehensive system for extracting temporal information from clinical texts was proposed by Tang et al. [20]. Tao et al. presented a method for identifying temporal representations of vaccine adverse events using ontology for temporal analysis [21]. Li & Patrick [22] addressed a statistical model using linguistic, contextual and semantic features for extracting temporal expressions from an extremely noisy clinical corpus. Xu et al. [23] introduced an end-to-end temporal relation system including a temporal extraction sub-system based on a Conditional Random Fields (CRF) for name entity extraction and context-free grammar-based normalization. Luo et al. [24] extracted temporal constraints from the eligibility criteria texts of clinical trials using CRF. Chang et al. [25] proposed a hybrid method TEMPTING to identify temporal links among entities combining a rule-based method and a maximum entropy model. Nevertheless, comparing and evaluating the performance of the systems could be difficult due to lack of system open source codes. Moreover, only a few of these systems processed complexity clinical texts, such as user-generated clinical notes. Most of them still focused on the process of the relatively formal clinical texts.

Moreover, there was research on Chinese temporal expression extraction. Li et al. [26] proposed a Chinese temporal tagging (extraction and normalization) method by developing Chinese HeidelTime resources. Shen et al. [27] constructed a temporal expression extraction model based on Tsinghua Chinese Treebank. Zhou et al. [28] proposed a method for the recognition of Chinese temporal expressions using regular expression matching and a temporal relationship extraction approach based on CRF. Yan and Ji [29] proposed a Chinese temporal information identification method using CRF and a semi-supervised learning method. Wu et al. [30] built a Chinese temporal parser for the extraction and normalization of temporal information utilizing grammar rules and constraint rules. Zhu et al. [31] presented a CRF-based approach for temporal phrases recognition. Liu et al. [32] proposed a new Chinese time expression recognition method combined with common features plus semantic role features according to the characteristics of Chinese time expression and CRF.

In the standardization of temporal annotation, TimeML is a robust specification markup language for annotating temporal expressions in texts [33]. It deals with four different issues in labelling temporal and event expressions including time stamping of events and reasoning with contextually underspecified temporal expressions. With respect to TimeML specifications [34], we use TIMEX3 for annotating temporal expressions throughout the paper. All the attributes of TIMEX3 are inherited from the THYME annotations [18].

## Methods

Aiming at automatically identifying temporal expressions from narrative multi-lingual clinical texts, TEER is proposed by leveraging temporal features, heuristic rules and automated learned patterns. In TEER, a temporal expression is represented as a triple *TE* = <*M*, *A*, *N*>, where *M* is a collection of temporal mentions, *A* represents the type attributes of the mentions *M*, and *N* is the normalized temporal values of the mentions *M*. The normalized values represent the temporal semantics of temporal expressions as specified using TimeML. Therefore, the purpose of TEER is to: 1) identify temporal mentions *M*, 2) assign *M* with type attributes *A*, and 3) normalize the values of mentions *M* into formal representations *N*.

**Fig. 1:**
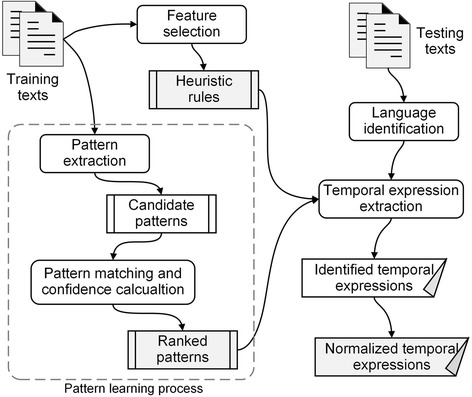
The overall framework of TEER incorporating heuristic rule generation and pattern learning

Initially, we summarize a list of heuristic rules representing a group of selected temporal features observed from temporal expression annotations for matching temporal expression candidates. After that, an algorithm is applied to automatically generate temporal structural patterns from clinical texts containing temporal expression annotations. Then the learned candidate patterns are matched to original texts for calculating matching confidence scores. The patterns evaluated by comparing with a confidence threshold are ranked and kept as final patterns. Eventually, the heuristic rules and the ranked patterns are combined to be applied to temporal extraction for newly coming clinical texts. The overall framework of the TEER is shown as Fig. 1.

In addition to English clinical texts. Temporal expressions in Chinese EMR have certain special representation features. We extract temporal expressions from manually annotated datasets and observe their features. Table 1 shows part of the identified features from training dataset. We further summarize a list of heuristic rules by analyzing the features of a list of training temporal expressions with human annotations. These heuristic rules in Chinese and English, as shown in Table 2, are used to identify temporal expressions for a given new clinical text.

**Table 1:** A list of identified temporal features in Chinese as examples

Types		Features
Unit		*年, 月, 日, 天, 周期, 星期, 周, 小时, 时, 分钟, 秒, 毫秒..*
Numeric		*半, 一, 二, 三, 四, 五, 六, 七, 八, 九, 十, 百, 千, 万, 兆, 亿*
Explicit	Day	*年-月-日, 月-日, 日..*
	Day-time	*上午, 下午, 早上, 白天, 傍晚, 晚上..*
	Week	*星期一, 星期二, 星期三, 星期四, 星期五, 星期六, 星期日, 星期天..*
	Month	*一月, 正月, 二月, 三月, ….*
Referred	Current	*现在, 现, 今, 今日, 当日, 本年, 今年, 本月..*
	Current-referred	*昨日, 昨天, 明日, 去年, 前年, 上年, 明年, 上个月, 下个月*
	Other-referred	*x后y天, x前y星期..*
Frequency		*第, 每..*
Implicit		*大概, 近期, 近, 约, 前, 后, 余前, 余, 来…*

**Table 2:** Examples of generated heuristic rules from Chinese clinical texts

Language	Expression	Representation format	Heuristic rule
Chinese	*约10天*	*[SLOT: prefix][SLOT: num][SLOT: day]*	*(‘+prefix+’)(\d{1,6})(‘+ dmy+’)*
	*2014年10月5日*	*[SLOT: num][SLOT: year][SLOT: num][SLOT: month][SLOT: num] [SLOT: day]*	*(\d{1,4}[年]\d{1,2}[月]\d{1,2}[日])*
	*约一个周期*	*[SLOT: prefix](LIST: 半|一|二|两|三|四|五|六|七|八|九|十|十一|十二|十三|十四|十五|二十|三十) < S: 个 > [SLOT: day]*	*(‘+prefix+’)(‘+num+’)(个)(‘+dmy+’)*
	*第7、9、14天*	*[SLOT: prefix][SLOT: num] < SM: 、 > [SLOT: num] < SM: 、 > [SLOT: num][SLOT: day]*	*(‘+prefix+’)(((\d{1,6})(‘+u’、‘+’)){1,5}(\d{1,6})(‘+dmy+’))*
English	*2014–11-04*	*[SLOT: year] < SM: - > [SLOT: month] < SM: - > [SLOT: day]*	*d{1,4}) r’(− |-| -|- |*)(*‘+[month] + r’(− |-| -|- |*)\ *d{1,2}*
	*November 2nd, 2016*	*[SLOT: month] < SM: >[SLOT: day](LIST: st, nd, rd, th) < SM:,> < SM: > [SLOT: year]*	*(‘+[month]+’(− |-| -|- |*)\ *d{1,2}(st|nd|rd|th)(, |,|*)\ *d{1,4})*
	*May 3–5, 2013*	*[SLOT: month] < SM: >[SLOT: day] < SM:- > [SLOT: day] < SM:,> < SM: > [SLOT: year]*	*(‘+[month] + r’ \d{1,2}(− |-| -|-*)\ *d{1,2}(, |,|*)\ *d{2,4})*
	*37 years old*	*<RULE: negative>*	*If context_distance([SLOT: num], [SLOT: age]) < n; Then filter out*

Using solely heuristic rules may cause incorrect matching. For example, “*1/17*” in “*左腋窝淋巴结(1/17)*” is wrongly matched by a heuristic rule as it is similar to a date representation “*January 17*”. Moreover, in clinical texts, some temporal expressions may be contextually connected, e.g., “*from Feb. 2016 to May 2016*”. A list of contextual relationships thus can be defined as patterns for extracting these types of temporal expressions. However, the summarized patterns without effective validation can cause wrong identifications of temporal expressions, e.g., the incorrect extraction of the ICD9 codes “*321.0*” and “*322.9*” in “*unspecified meningitis 321.0–322.9*” as temporal expressions. Consequently, an algorithm incorporating with automatic temporal pattern learning is more preferred.

Our method TEER automatically generates a group of temporal patterns from a manually annotated training dataset and validates the generated patterns. The core idea of the pattern learning is to extract all pattern candidates with high accuracy confidence in matching original annotated texts. Those patterns with a confidence lower than a threshold are filtered out and the patterns are applied to verify temporal expression candidates that cannot be determined by solely heuristic rules. Finally, TEER normalizes the extracted temporal expressions and represented them using TimeML.

As shown in Algorithm 1, the detailed automatic pattern learning algorithm mainly contains the following five steps: 1) sentence boundary detection. This is to split discharge summary texts into sentences. We use both the widely used NLTK sentence boundary identifier and our defined rules to correct the splitting since some incorrect sentences may be obtained such as “e.g.*,*” symbols; 2) tag replacement. This is to replace the original annotation temporal expression tags with a specified tag for the purpose of context extraction; 3) extract candidate patterns. By setting a word window length, we can extract all possible candidate patterns that containing the specified tag and their surrounding words, as shown from line 7 to 11. We empirically choose 8 as the word window size; 4) calculate pattern confidence. Each candidate pattern is matched back to original texts to calculate its support and confidence values. Support metric is defined as the count of correct matches and confidence metric is defined as the rate of correct expression matches among all matches; 5) final pattern generation. By comparing with a confidence threshold, the candidates with confidence greater than or equal to the threshold are kept as final learned patterns.

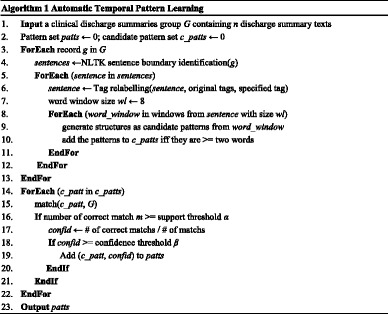



For example, there is a sentence “*入院后完善相关检查,未见明显手术禁忌症,遂于* < T > *22/9*</T > *行右乳癌改良根治术 + 右腋窝前哨淋巴结活检 + 腋窝清扫术,术后病理未回*”, where “<T > </T>” is a temporal annotation tag defined by annotators. Our algorithm replaces the whole temporal expression annotations with a [TE] tag. With the setting of word window length, a list of pattern candidates are extracted, as shown in Table 3. After that, all the pattern candidates are matched to all training texts to calculate their support and confidence values. For example, “*于*[TE]*行*” is matched with “*于* < T > *2014–9-30*</T > *行右乳癌根治性保乳术*”, “*于* < T > *2014年10月5日*</T > *行腹腔镜探查术*”, and “*于门诊行*”. Only those candidate patterns with both support and confidence values larger than predefined thresholds are considered as validated patterns for applying to a testing dataset. We empirically set support threshold *α* = 3 and confidence threshold *β* = 0.6 to enlarge their matching coverage.

**Table 3:** A list of pattern candidates with their support and confidence values generated by the algorithm for the example sentence

Pattern candidates	Support	Confidence	Keep
遂于[TE]行右乳癌	3	1	YES
遂于[TE]行右	3	1	YES
遂于[TE]行	4	1	YES
遂于[TE]	5	0.83	YES
于[TE]行	8	0.4	NO
于[TE]	34	0.52	NO
[TE]行	9	0.06	NO

Eventually, the extracted temporal expressions need to be formally represented. Based on our previous experience on English clinical text annotation [1], we continually apply the temporal expression schema TimeML and use TimeX3 as the annotation format standard. Using the markup language, a temporal expression can be annotated in a XML-based format, e.g., <TIMEX3 tid = “*t5*” Type = “*Duration*” Value = “*P3D*” > *3 days*</TIMEX3>. In the TimeX3 representation, each expression is labelled with one of the four types: “Date”, “Time”, “Duration”, and “Set”. The representations of the types are formally defined in the TimeX3. For example, the “Duration” type starts with letter ‘*P*’ as duration usually denotes a period of time. In TEER, we classify the extracted temporal expressions into the types and normalize them using commonly used normalized value representations. For example, “<T > *今日*</T > *出院*” is labelled and normalized as “<TIMEX3 tid = “*t2*” Type = “*Date*” Value = “*2014–10-7* ” > *今日*</TIMEX3>*出院*”, where “*Date*” is a type and “*2014–10-7* ” is the normalized value of “*今日*” (*today*) corresponding to its reference date of “*出院*”(*discharge date*).

## Results

### Datasets

For English clinical texts, we used the same 400 real free-text clinical requests, as used in [1], submitted to the data warehouse of Columbia University Medical Center (CUMC). Three human annotators including two clinical researchers and one IT scientist labelled all the request texts. The overlap among the annotators was calculated as 87%. All the disagreements were recorded and further manually classified as “minor”, “partial”, and “distinct”. Most of the disagreements (92%) were minor (e.g., “*the* < T > *last month*</T>” versus “<T > *the last month*</T>”) and partial (e.g. “*through* < T > *May 11 = 02*</T>” versus “*through* < T > *May 11*</T > *=02*”). All the disagreements were eventually resolved through discussions. All annotations followed the TimeML annotation guideline. Among the 400 annotated request texts, 100 were randomly selected as a training dataset and the remaining 300 requests were used as a testing dataset. The 400 request texts contained 1044 sentences (2.61 sentences per request on average) and 2397 tokens in total. There were in total 553 temporal expressions identified and annotated (1.38 temporal expressions per request text on average).

For Chinese clinical texts, the EMR records of 1459 real patients with breast cancer, acquired from a 3A hospital in mainland China, were used. We randomly selected 400 narrative discharge summary texts considering manageable human annotation workload with 276 as a training dataset, and 134 as a testing dataset. The 400 EMR texts contained 11,943 sentences (29.86 sentences per record on average) and 35,250 clauses in total. A total of 5303 temporal expressions were annotated (13.26 temporal expressions per record text). Please note each temporal expression had been labeled with date type, e.g., <T Type = “*Time*” > *2014/10/8 9:16:34*</T>, <T Type = “*Date*” > *5月26日*</T>, <T Type = “*Duration*” > *一个月*</T>, <T Type = “*Set*” > *第7、9、14天*</T>, etc. A summary of the two datasets is reported in Table 4.

**Table 4:** The data summary of the training and testing datasets

Language	Dataset	#texts	#sentences	#clauses	#temp. Exp.	#ave. temp. exp. /text
Chinese	Training	276	7747	23,423	3525	12.77
	Testing	134	4196	11,827	1778	13.27
	Total	400	11,943	35,250	5303	13.26
English	Training	100	257	694	155	1.55
	Testing	300	787	1703	398	1.33
	Total	400	1044	2397	553	1.38

### Baselines

We use the following six publicly available temporal expression extraction systems as baseline methods for performance comparison:

1. HeidelTime [35] is a domain-sensitive temporal expression tagger supporting multi-languages developed by the Dataset Systems Research Group at Heidelberg University. It uses a group of rules and adopts different strategies, according to the domain texts that are to be processed. It utilizes hand-crafted resources and currently supports 13 languages including Chinese. It is used to automatically create resources for more than 200 languages.
2. IllinoisTemporalExtractor (The Illinois Temporal Extractor) [36] extracts temporal expressions as well as optionally associate the expressions to reference dates. The method can be applied programmatically. A number of rudimentary command-line functions are provided to enable users reuse and evaluate its capability locally. It also provided an online version. The method currently works for English plain texts only.
3. GUTime [37] utilizes the TimeML TIMEX3 as the standard for temporal expression annotation. It extends TempEx and allows using functional styles of encoding offsets in temporal expressions. The method also can handles a variety of temporal expressions that are not covered by TempEx, including varieties of temporal modifiers and date formats in different countries.
4. SUTime [38] is a part of the Stanford CoreNLP pipeline. It uses a rule-based temporal tagging strategy to annotate temporal information in free texts. SUTime outperformed a list of baseline methods on the TempEval-2 evaluation challenge.
5. NLTK Timex [39] is a library belongs to NLTK Contrib repository. It contains a module named as timex.py for identifying temporal expressions. The Timex library is open source and widely applied to general natural language processing tasks. The library provides a general way to annotate and normalize English temporal expressions.
6. CMedTEX [40] is a rule-based system for extracting and normalizing temporal expressions for Chinese electronic medical records. It can identify four types of temporal expressions including Date, Time, Duration and Frequency. It normalizes temporal values with TIMEX3 standard. The CMedTEX has been applied to annotate Chinese clinical texts and has reported a good performance of temporal expression extraction and normalization.

### Results

Based on the two training datasets, TEER generated 21 heuristic rules and 65 structural patterns in total for English data request texts, while generated 26 heuristic rules and 58 structural patterns for Chinese EMR record texts. Table 5 shows some example patterns with an example of their matched texts.

**Table 5:** The examples of learned patterns that are categorized into four types

Language	Type	Structural pattern	Matching text
English	Time	with [TE] prior to	*starting with 24 h prior to the patients admission*
	Date	discharged in [TE]	*surgery patients discharged in 2015*
	Duration	study period [TE] - [TE]	*study period May 1st, 2016 - May 21th, 2016*
	Set	issued on a [TE] basis	*issued on a weekly basis*
Chinese	Time	后[TE]内	*在入院后 24小时内*
	Date	[TE]返院化疗	*2014.11.3返院化疗*
	Duration	[TE]/次	*3–5天/次*
	Set	[TE]检查	*每三个月检查一次*

In the evaluation, CMedTEX had a different temporal annotation standard with us on Chinese clinical texts. For example, it annotated “*后1年余*” as “*后* < T > *1年余*</T>” while our method annotated it as “<T > *后1年余*</T>”. To compare the methods with the same annotation standard, we intentionally revised our TEER method to adapt to the annotation standard of CMedTEX. For example, “[*temporal expression*] *后*” and “*后* [*temporal expression*]” were labeled as “<T > [*temporal expression*]</T > *后*” and “*后* < T > [*temporal expression*]</T>”, respectively. We named the revised method as TEER_C, which was used for Chinese clinical text processing only.

**Fig. 2:**
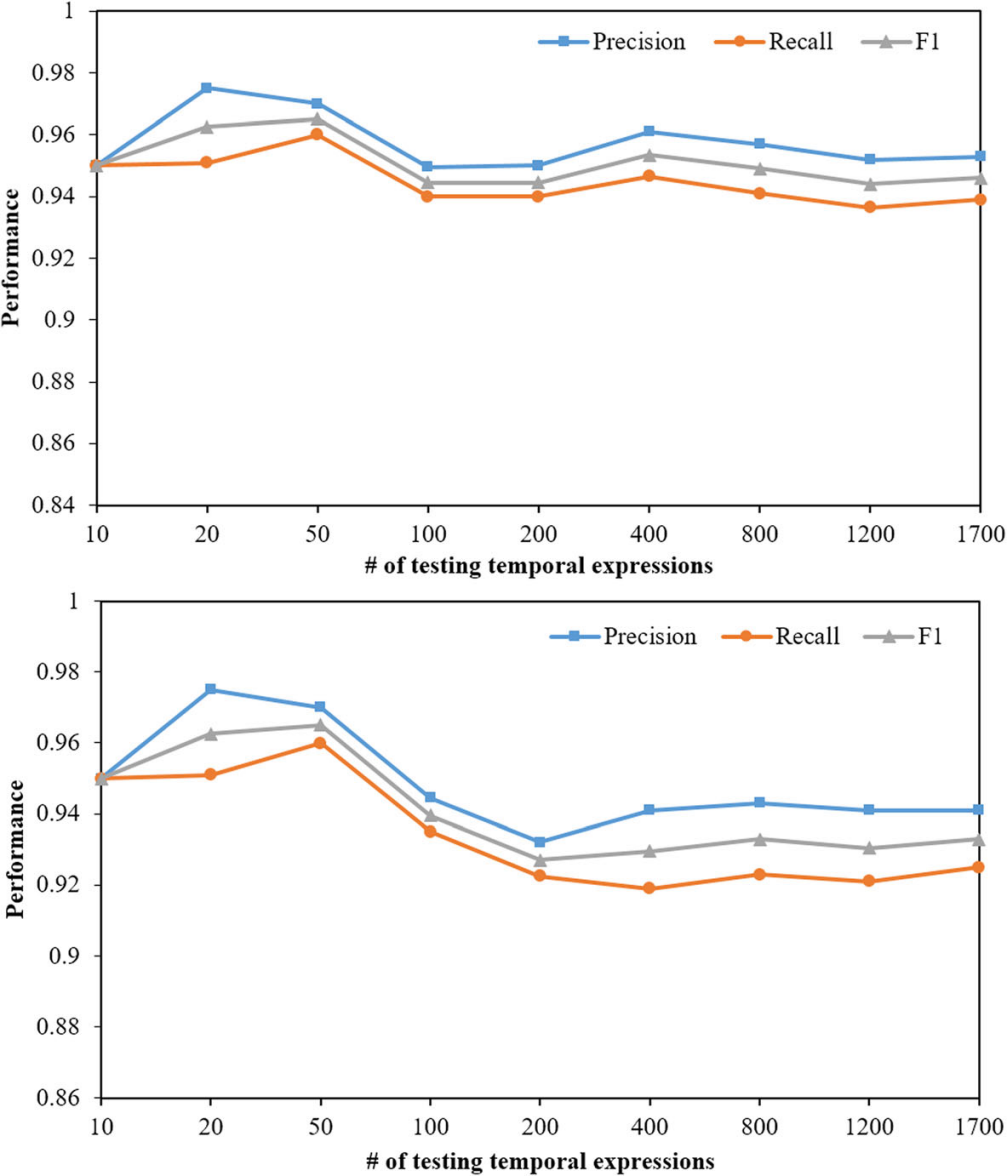
An implemented graphical user interface of TEER for temporal expression extraction

After that, we applied both TEER and TEER_C to test the scalability of our method on various numbers of temporal expressions randomly selected from the training dataset. We tested a different number of expressions from the Chinese EMR record text data with the increasing number from 10 to 1700. The results, as shown in Fig. 2, presented that the performances of both TEER and TEER_C were relatively stable when the number of temporal expressions was larger than 200. After that, the performance changed slightly in terms of F1 score with the increasing number of testing temporal expressions.

After training TEER and TEER_C on the two training datasets, all the testing datasets containing 1778 Chinese temporal expressions and 398 English expressions were applied to each of the baseline methods for performance comparison. The results are shown as Table 6. For Chinese text annotation, GUTime had a F1 score of 0.375 only, while Heideltime had a F1 score of 0.603. Though CMedTEX obtained a lower precision as 0.717, it significantly improved the recall to 0.925, achieving an overall F1 of 0.808. Our method TEER_C achieved a recall of 0.907 while a higher precision of 0.916, increasing the F1 to 0.912. The TEER further improved the performance into a F1 of 0.936. For English text annotation, NLTK Timex, GUTime, and IllionisTExtractor acquired F1 scores as 0.242, 0.353, and 0.765, respectively. Heideltime obtained a precision of 0.828 and a recall of 0.822. One of our previous work named as TEXer, as reported in [1], achieved a F1 score of 0.897. Our TEER achieved a precision of 0.948, a recall of 0.877, and a F1 score of 0.911, outperforming all the baseline methods.

**Table 6:** The performance comparison of our method with baseline methods

Language	Method	# exp.	# correct	Precision	Recall	F1
Chinese	GUTime	512	429	0.838	0.241	0.375
	HeidelTime	954	824	0.864	0.463	0.603
	CMedTEX	2293	1645	0.717	0.925	0.808
	TEER_C	1761	1613	0.916	0.907	0.912
	TEER	1761	1657	0.941	0.932	0.936
English	NLTK Timex	106	61	0.575	0.153	0.242
	GUTime	101	88	0.871	0.221	0.353
	IllinoisTExtractor	352	287	0.815	0.721	0.765
	Heideltime	395	327	0.828	0.822	0.825
	TEXer	360	340	0.944	0.854	0.897
	TEER	368	349	0.948	0.877	0.911

## Discussion

We analyzed all extraction errors by our TEER and the baseline methods. For the English temporal expression extraction, some errors were brought by special clinical codes in the clinical data requests in the representation of numerical values, e.g., ICD9, CPT codes, etc. For example, “*between 2005 - 2007 with one of the following DRG codes 291.0 Alcohol Withdrawal, 303.0 Acute Alcoholic Intoxication, or 305.0 Alcohol Abuse*”. NLTK Timex labelled most of the codes as temporal expressions incorrectly, dramatically lowering its performance. The requests also contained a large number of quantitative expressions, which frequently caused errors using NLTK Timex, GUTime, and IllionisTExtractor. For example, IllinoisTExtractor incorrectly annotated “*2000*” as a date in “*2000 mg per ml*”. Moreover, some user-defined temporal expressions with special representation format frequently caused unexpected errors. For instance, the texts “*May 22 = 02*” and “*patient started HD 2/00*” were in uncommon representation formats. As a result, TEER and the baseline systems only labelled them partially. Finally, noise data, such as incorrect spellings affected the performance of the methods, e.g., “*1995-pr!esent*” (“*present*” was expected) and “*2 moths*” (“*months*” was expected). The processing of clinical request texts commonly suffered from the above types of errors. Therefore, the design of new methods need to tolerate the texts as much as possible. TEER showed improvement on this particular point.

In addition to the errors caused by the complexity of the texts, some errors were incurred by the methods themselves. The GUTime heavily relied on Part-of-Speech tagging thus it incorrectly annotated some texts. For example, the “*2/3/1999*” was incorrectly tagged into “2*/3/* <TIMEX3 tid = “*t1*” Type = “*Date*” Value = “*1999*” > <lex pos = “*CD*” > *1999* </lex></TIMEX3>”. The method incorrectly tagged “*the current*” as EVENT type rather than temporal expression based on POS tags. The Heideltime wrongly ignored some special temporal terms and abbreviations, e.g., “current”, “weekly”, and “*12 h*”. The IllinoisTemporalExtractor incorrectly labelled all “*fall*” words as temporal expressions but none was truly temporal expression, e.g., “*falling down*”. The method also missed some short representations, e.g., “*present*”, and minute type, e.g., “*2 mins*”. NLTK Timex performed worst on the identification of month and year abbreviations, e.g., only “*2015*” was identified in “*Feb. 2015*”. Moreover, all the baseline methods labelled age-related expressions as temporal expression, while the age information should be independently annotated and distinguished from temporal information. TEER had errors on the identification of some certain abbreviations. For instance, it incorrectly annotated “*MAR*” as a date type in “*MAR info tables*”.

For the Chinese temporal expression extraction, GUTime only identified two types of temporal expressions: Date and Time. According to our evaluation, it was not suitable for Chinese clinical texts due to its very limited language-specified rules. Heideltime correctly identified most of common and simple temporal expressions but it missed complex expressions. For example, it tagged “*5月*” in “*5月余*” and “*1月*” in “*1月后*” only and wrongly missed their modifiers “*余*” and “*后*”. CMedTEX correctly extracted these frequently used expressions such as “*三次*” and “*随诊*”. However, it incorrectly tagged hospital admission numbers and patient ages as temporal expressions. For example, the “*1258年*” in “*住院号: 761258 年龄:52岁*” was annotated as a temporal expression. In addition, CMedTEX incorrectly tagged some of the mentioned medical codes (e.g., ICD9, ADT, and CPT codes) as temporal expressions. In TEER, the learned patterns were able to use contextual relations to eliminate the interference of these codes. From the results, the pattern learning was tested to be an effective strategy in temporal expression identification.

Nevertheless, we identified a number of error cases by TEER method. The errors and their potential reasons were summarized as follows: 1) Errors by heuristic rule and pattern coverage. Since the heuristic rules and patterns were either summarized or automated learned from the limited number of annotated training dataset, some necessary rules and patterns were missing for the testing dataset. For example, a new temporal expression “*1 + 月*” was found in the testing dataset but was incorrectly annotated due to the lack of matched patterns. 2) Errors caused by informal representations or typos. Clinical discharge summary texts contained a number of informal representations or even typos, causing unexpected errors. For example, the text “*2013..07*” contained a punctuation typo “*..*” and “*2014–09-2014/10/11*” was an complex informal representation, causing wrong annotations by TEER. These informal cases and typos were unpredictable and inevitable as they may come from clinicians themselves.

**Fig. 3:**
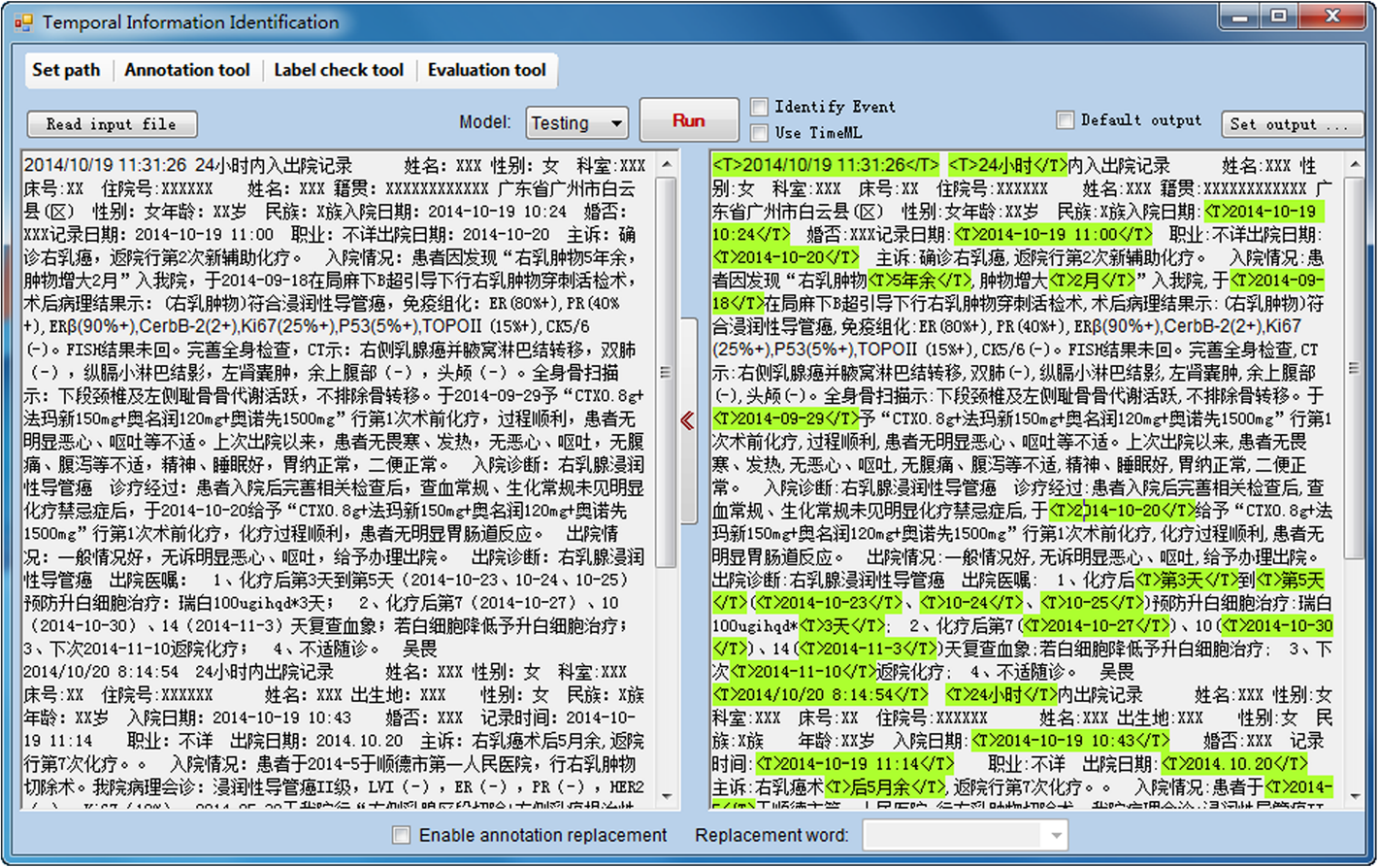
The scalability evaluation of TEER (the first) and TEER_C (the second) using the increasing number of temporal expressions randomly selected from the testing datasets

The TEER method was programmed using Python and a graphical user-interface was implemented using C#. As shown in Fig. 3, TEER can annotate the raw texts in the left side panel automatically and output the annotated results in the right side panel. The identified temporal expressions are marked in green color, starting with “<T>”. Users can select the “Use TimeML” option to output results in TimeML annotation format. In addition to the TimeX3 format, it also supports other types of annotation formats including user specifically defined annotation tags. The annotation output is visualized using different colors, where green color denotes temporal expressions and bule color denotes events. The system also provides a tool for quick manual annotation on raw texts as well as a standard evaluation tool for comparing the system output with gold stand annotations or the outputs from other baseline methods. TEER can be used in the ways of graphical user-interface, library integration, Web-based, and command line-based interface. TEER can be potentially applied to a variety of natural language processing libraries for clinical text processing. The python code was publically available on Github (https://github.com/Tony-Hao/TEER).

## Conclusions

Temporal expression extraction is a fundamental and essential step in the processing of clinical texts. This paper proposed an automated method TEER for multi-lingual temporal expression extraction through the learning of temporal features, heuristic rules, and patterns. Based on the two datasets including 400 actual clinical data requests in English and 1459 EMR records in Chinese, we conducted the comparison of TEER with six baseline methods. The experiment results presented that the TEER method achieved the best performance, demonstrating its effectiveness in the temporal expression extraction from multi-lingual heterogeneous clinical texts. We will continue to improve the performance of TEER on temporal abbreviation identification, temporal value normalization, and event identification in the future.

## Abbreviations

ADT: Admit Discharge Transfers
CPT: Current Procedural Terminology
CRF: Conditional Random Fields
CUMC: Columbia University Medical Center
EMR: Electronic Medical Record
i2b2: Informatics for Integrating Biology & the Bedside
ICD: International Classification of Diseases
IllinoisTemporalExtractor: The Illinois Temporal Extractor
NLP: Natural Language Processing
TEER: Temporal Expression ExtractoR
