# Correction to: A pattern learning-based method for temporal expression extraction and normalization from multi-lingual heterogeneous clinical texts

**DOI:** 10.1186/s12911-018-0603-0

**Published:** 2018-04-13

**Authors:** Tianyong Hao, Xiaoyi Pan, Zhiying Gu, Yingying Qu, Heng Weng

**Affiliations:** 1 School of Information Science and Technology, Guangdong University of Foreign Studies, Guangzhou, China; 2 School of Computer, South China Normal University, Guangzhou, China; 3 School of Business, Guangdong University of Foreign Studies, Guangzhou, China; 4 The Second Affiliated Hospital, Guangzhou University of Chinese Medicine, Guangzhou, China

## Erratum

After publication of the original article [1] it was noted that the captions relating to Figs. 2 and 3 had been interchanged.

**Fig. 2:**
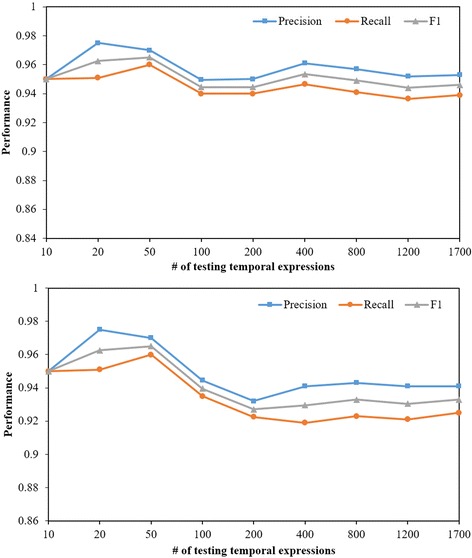
An implemented graphical user interface of TEER for temporal expression extraction

**Fig. 3:**
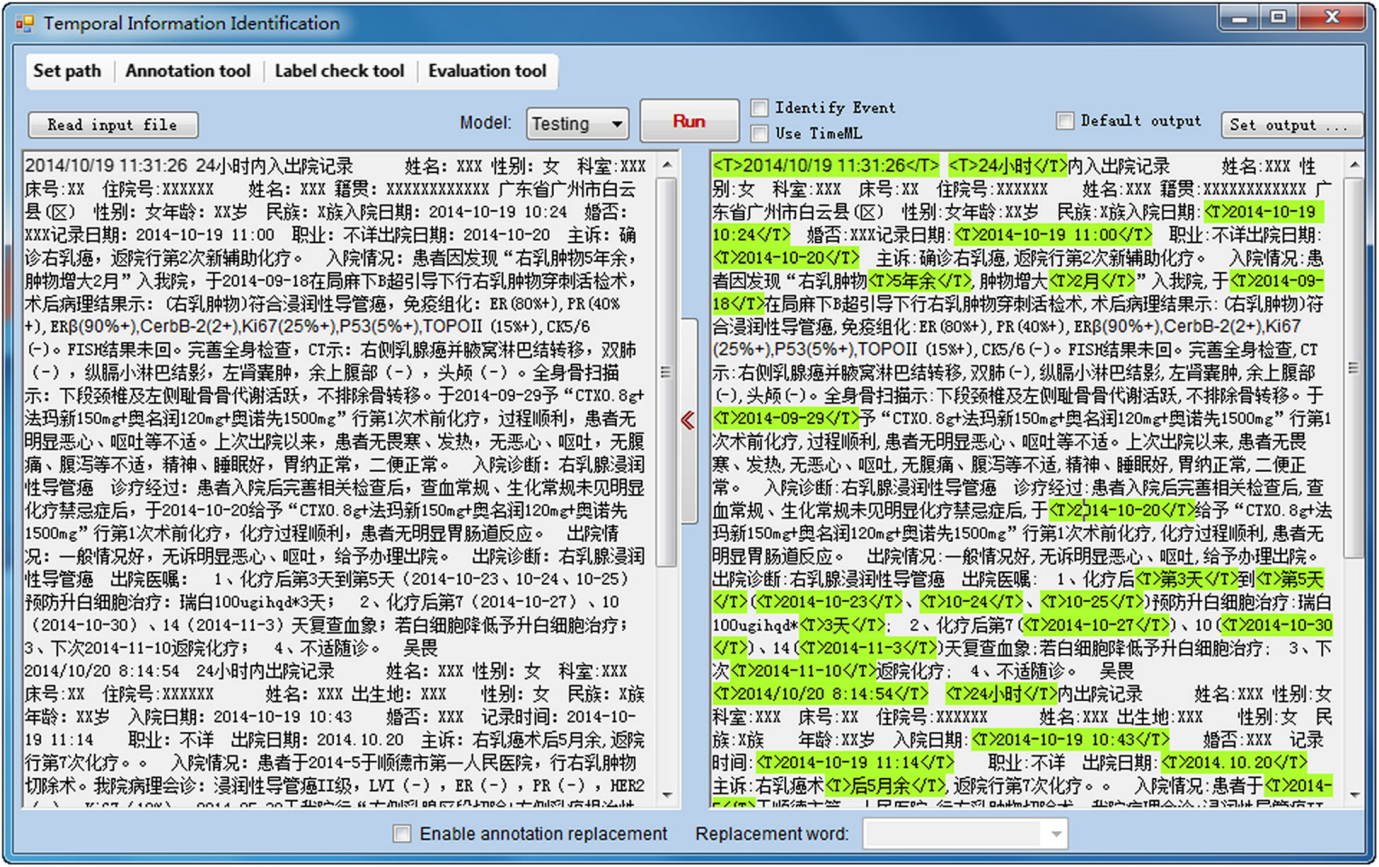
The scalability evaluation of TEER (the first) and TEER_C (the second) using the increasing number of temporal expressions randomly selected from the testing datasets

These errors were introduced during typesetting; thus the publisher apologizes for this error. The correct figures and corresponding captions are shown below.
